# Rheumotologitsts’ view on the use of hydroxychloroquine to treat COVID-19

**DOI:** 10.1080/22221751.2020.1760145

**Published:** 2020-05-02

**Authors:** Xiaoxuan Sun, Yicheng Ni, Miaojia Zhang

**Affiliations:** a Department of Rheumatology, The First Affiliated Hospital of Nanjing Medical University, Nanjing, People’s Republic of China; b Department of Imaging & Pathology, Faculty of Medicine, KU Leuven, Belgium

**Keywords:** COVID-19, SARS-CoV-2, therapy, hydroxychloroquine (HCQ), Chloroquine (CQ)

## Abstract

The current pandemic coronavirus disease 2019 (COVID-19) caused by the severe acute respiratory syndrome coronavirus 2 (SARS-CoV-2) calls urgently for effective therapies. Anti-malarial medicine chloroquine (CQ) and particularly its chemical analogue hydroxychloroquine (HCQ) have been recommended as promising candidate therapeutics that are now under either compassionate off-label use or clinical trials for the treatment of COVID-19 patients. However, there are public concerns and disputes about both the safety and efficacy of CQ and HCQ for this new application. Given the fact that for decades HCQ has been approved as an immunomodulatory drug for the long term treatment of chronic rheumatic diseases, as experienced rheumatologists, we would like to share our thoughts in this regard and trigger a brainstorm among clinical care providers for exchanging their diverse opinions on this urgent topic.

## Introduction

The current outbreak of coronavirus disease 2019 (COVID-19) caused by the severe acute respiratory syndrome coronavirus 2 (SARS-CoV-2) poses a serious threat to global public health and local economies [1]. Such huge numbers of infected people and deaths call for an urgent and effective therapy to treat symptomatic patients, which can also decrease the duration of virus carriage in order to limit the transmission of viruses in the community.

As originally an anti-malarial medicine applied for decades, hydroxychloroquine (HCQ) is one of the disease-modifying antirheumatic drugs (DMARDs), which is widely used for treating certain rheumatic diseases such as rheumatic arthritis (RA) and systemic lupus erythematosus (SLE), and it also demonstrates a strong immunomodulatory capacity, which prevents inflammation flare-ups and organ damage. Some clinicians recommend that patients with COVID-19 should be counselled about HCQ treatment [2 31–3]. However, the optimal dosage, course and the side-effect for HCQ in SARS-CoV-2 infection need to be assessed by the standard clinical trials in the coming days.

## HCQ may confer an antiviral effect at early infection stage

Chloroquine (CQ) is a drug widely used in the treatment of malaria and autoimmune diseases, reportedly having broad-spectrum antiviral potential [4]. Given that HCQ demonstrates similar molecular mechanisms to CQ, it is highly likely that HCQ will perform similarly in terms of early prevention of disease progression [5]. The antiviral activity of HCQ may be due to the following four ways [6]: (1) reducing the angiotensin-converting enzyme 2 (ACE2) receptor terminal glycosylation on the surface of cells thus interfering with the binding of SARS-COV-2 to the ACE2 receptor; (2) increasing the pH of endosomes and lysosomes to prevent the fusion process of the virus with host cells and subsequent virus replication; (3) preventing antigen processing and major histocompatibility complex (MHC) class II-mediated autoantigen presentation to T cells, which reduces T cell activation, differentiation and expression of co-stimulatory proteins and cytokines produced by T cells and B cells (e.g. IL-1, IL-6 and TNF-α); and (4) disrupting the interaction of DNA/RNA with toll-like receptors (TLRs) and the nucleic acid sensor cyclic GMP-AMP (cGAMP) synthase (cGAS) and therefore the transcription of pro-inflammatory genes cannot be stimulated [6]. It is speculated that HCQ functions at entry of the SARS-COV-2 infection in Vero E6 cells [7]. Antiviral drugs should be used to reduce viral replication in the early stages of disease based on the dynamic results of clinical trials. Gautret et al. reported that azithromycin (AZM) added to HCQ was significantly more effective for virus elimination than using HCQ alone. They used 200 mg of HCQ three times a day for 10 days, plus AZM if deemed necessary. A higher frequency of COVID-19 clearance was noticed after 6 days of treatment with HCQ alone or HCQ + AZM versus the untreated control group (70% vs 12.5%; *P*<0.001) [2]. In our opinion, patients before having shortness of breath or in the high-risk category even with just mild symptoms should be treated as soon as possible.

The transition to Acute Respiratory Distress Syndrome (ARDS) occurs in many severe COVID-19 cases because of the cytokine release syndrome (CRS), or “cytokine storm”[8]. Guastalegname and Vallone controversially commended that to the SARS-CoV-2 positive patients, there might be no benefits of HCQ, if not any harm, on mortality and lymphopenia improvement [9]. Immunosuppressants should be used with caution to combat cytokine storms and reduce mortality in severe patients.

## Safety and adverse events

HCQ exhibits better anti-SARS-CoV-2 activity and fewer toxic side effects than CQ [1,8]. Gautret et al. showed that HCQ (200 mg, three times per day for ten days) is efficient in clearing viral nasopharyngeal carriage of SARS-CoV-2 in only three to six days, in most COVID-19 patients [2]. A recent Chinese study has recommended that HCQ sulfate can be given orally for SARS-CoV-2 infected patients at the dose of 400 mg twice daily for the first day, followed by a maintenance dose of 200 mg given twice daily for 4 days [3]. Expert consensus statement has recommended HCQ in an attempt of antiviral treatment in China [10].

Long-term exposure of the patients to HCQ can cause side effects such as retinopathy and cardiomyopathy. Although HCQ has a low level of tissue accumulation, high-dose and long-term (over 5 years) intake of HCQ is likely to contribute to the development of retinopathy [11]. However, a study that focused on the dose loading with HCQ (400, 800 mg/day, and 1,200 mg/day groups) for RA patients showed that ocular abnormalities occurred in 17 of 212 patients (8%), and there were no adverse ocular events at 6 weeks [12]. Luckily long-term treatment is not needed for COVID-19 patients.

Previous study also suggested a synergistic effect of the combination of HCQ and AZM [8]. Speculated potential risk of severe QT prolongation induced by the association of the two drugs and viral myocarditis caused by SARS-CoV-2 infection should be considered. This side effect is rare, but it is not clear whether their combined use in the treatment of COVID-19 may amplify this risk. In this sense, HCQ should be avoided in patients with underlying cardiovascular diseases. Electrocardiogram (ECG) examination should be performed before the treatment, and ECG monitoring can be performed if necessary.

As rheumatologists, we have comprehensive experiences in successfully treating patients with chronic autoimmune diseases with long-term HCQ treatment (400 mg/day for more than 5 years). It was reported that the safe dosage (6–6.5 mg/kg per day) of HCQ sulfate could generate serum levels of 1.4–1.5 μM in humans [11]. HCQ has a low level of tissue accumulation, and the maximum tolerable dose for HCQ is 1200 mg [12]. Therefore, 400 mg per day for HCQ is in a safe dosage range. Patients with long-term exposure to HCQ at this safe dosage suffer from few side effects.

Importantly, the outbreak of SARS-CoV-2 has placed pregnant women at high risk of infection. HCQ should be considered as a potential therapeutic solution for these patients, given its safety profile in pregnancy [13,14].

## Summary

We suggest that HCQ has few side effects and should be used as an initial treatment as soon as the diagnosis of COVID-19 is made. When the disease progression is from mild to severe, hospitalization and stronger immunosuppressive treatment are required. Our opinions on HCQ/CQ for the treatment of COVID-19 here appear somewhat different from the more conservative ones expressed recently by other rheumatology colleagues [15]. The role of HCQ in against COVID-19 needs to be thoroughly evaluated in both observational studies and high quality randomized controlled studies.
